# Nanocarriers for Combination Therapy in Pancreatic Ductal Adenocarcinoma: A Comprehensive Review

**DOI:** 10.3390/nano15151139

**Published:** 2025-07-22

**Authors:** Iris Pontón, David Sánchez-García

**Affiliations:** Grup d’Enginyeria de Materials (GEMAT), Institut Químic de Sarrià (IQS), Universitat Ramon Llull (URL), Via Augusta 390, 08017 Barcelona, Spain; iris.ponton@iqs.url.edu

**Keywords:** pancreatic cancer, pancreatic ductal adenocarcinoma, nanoparticles, nanomedicine, combination therapy, multi-drug resistance, chemotherapy, stromal therapy

## Abstract

Pancreatic ductal adenocarcinoma (PDAC) remains one of the deadliest cancers worldwide, characterized by late diagnosis, aggressive progression, and poor response to conventional monotherapies. Combination therapies have emerged as a promising approach to overcome multidrug resistance (MDR), enhance efficacy, and target the complex tumor microenvironment (TME). Nanoparticle-based drug delivery systems (DDSs) have gained significant attention for their ability to co-deliver multiple agents with controlled release profiles. This review comprehensively examines nanoparticle-based platforms developed for PDAC combination therapies, focusing on small-molecule drugs. The systems discussed are drawn from studies published between 2005 and 2025.

## 1. Introduction

Cancer encompasses a diverse group of diseases arising from a multistage process that can originate in nearly any organ or tissue of the body, leading to the uncontrolled growth and spread of abnormal cells [1]. It is characterized by the accumulation of morphological and genetic alterations, caused by external (chemicals, radiation, infectious organisms) and internal factors (hormones, immune conditions, random mutations) [2]. Remarkably, it is a significant health issue in developed countries, where it is primarily associated with the aging population and their lifestyle [3]. According to the World Health Organization report from 2019, cancer is currently the second-leading cause of death worldwide [4,5]. In particular, pancreatic cancer is the fourth leading cause of cancer-related death in Europe and the United States and the most life-threatening cancer type worldwide. Despite representing only 3% of cancer incidence, it is responsible for 7% of all cancer-related deaths. This disproportionate impact is reflected in its dismal median overall survival, which is typically only 5 to 6 months [6,7]. This low survival is attributed to several factors, especially, the late diagnosis, as most patients remain asymptomatic until the disease progresses to advanced stages [8]. Pancreatic ductal adenocarcinoma (PDAC) is by far the most common pancreatic cancer, accounting for 95% of cases, and is one of the most aggressive solid malignancies, having become the third deadliest cancer in 2023 [9,10,11,12]. The 5-year survival rate is estimated to be less than 5%, and one-year survival is observed in less than 20% of cases [9,11].

PDAC originates in ductal cells, located at the exocrine part of the organ, which are responsible for transporting digestive enzymes. These cells, of epithelial origin, eventually undergo a process called epithelial-to-mesenchymal (EtM) transition. Briefly, the EtM transition involves a huge transformation where cells proliferate and exit the epithelial compartment, migrating and interacting with stromal cells producing the primary tumor [13].

The aggressiveness of pancreatic cancer is largely due to the dense and fibrotic stroma that surrounds the cancer cells, which not only acts as a physical barrier bur also promotes the malignancy of the tumor. Moreover, the leaky and abnormal vasculature, which tends to collapse, hinders drug accumulation and limits treatment efficacy [14].

From 1996 to 2011, Gemcitabine GEM (Gemzar^®^) was considered a gold-standard chemotherapy for treating pancreatic cancer [15]. However, chemo-resistance associated with this drug limits its effectivity [16]. To overcome this hurdle, combination therapies such as NALIRIFOX (liposomal irinotecan, oxaliplatin, fluorouracil, and leucovorin) have been proposed [12]. Unfortunately, despite significant medical advancements in the treatment of solid tumors over recent decades, the mortality-to-incidence ratio for PDAC remains stubbornly high [9].

However, in the last decade, nanomedicine has revolutionized PDAC therapy through nanoscale DDSs that enable controlled drug release. These systems surpass conventional therapies by offering sustained release, reduced systemic toxicity, enhanced stability, and increased drug concentration at tumor sites, improving efficacy and minimizing side effects. A key example is nab-paclitaxel (nab-PTX, Abraxane^®^), a ~130 nm nanoparticle conjugating paclitaxel with albumin, enhancing PTX solubility and tumor targeting via receptor-mediated uptake. Nab-PTX’s clinical success, with improved efficacy and lower toxicity, serves as proof of concept for PDAC nanomedicines [17]. Similarly, nal-IRI (Onivyde^®^), a ~111 nm liposomal nanoparticle encapsulating irinotecan, ensures prolonged circulation and tumor accumulation through the enhanced permeability and retention (EPR) effect, reducing toxicity while maintaining efficacy [18]. Nanomedicines like nab-PTX and nal-IRI highlight the potential of nanoscale DDSs to transform PDAC treatment, paving the way for selective and more effective PDAC therapies.

This review explores nanoparticle-based platforms developed for PDAC combination therapies, focusing on small-molecule drugs for monotherapy or in combination with other therapies. Nanocarriers designed for imaging applications [19], gene therapy, or stromal-targeted therapies [20] have been reviewed elsewhere in the literature.

## 2. Pancreatic Ductal Adenocarcinoma

**Figure 1:**
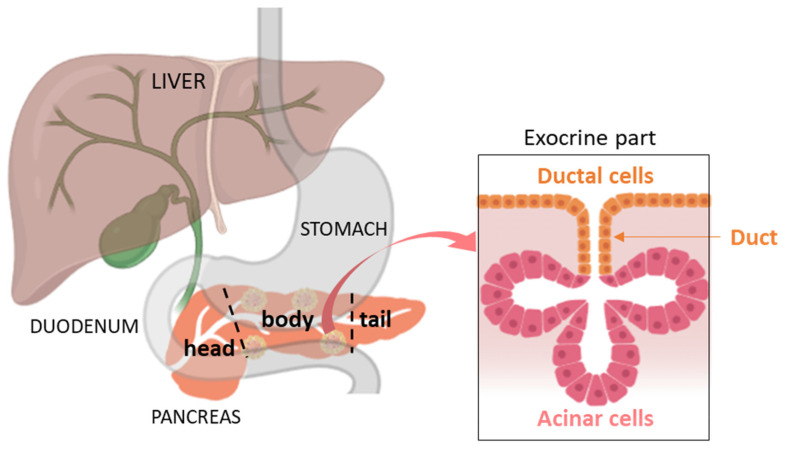
Graphic representation of the digestive tract, with the pancreas and its anatomical regions (head, body, and tail) highlighted. The exocrine portion of the pancreas is emphasized for clarity. Image created using BioRender.com.

The pancreas is a vital organ of the digestive system, comprising both exocrine and endocrine components [21]. The exocrine pancreas, which constitutes the majority of the organ’s volume, produces digestive enzymes, while the endocrine pancreas, accounting for only 1–2% of the organ, consists of specialized cells that secrete hormones essential for regulating blood sugar and other functions [22]. Approximately 80% of PDAC cases originate in the exocrine pancreas, with about 75% located in the pancreatic head (Figure 1) [11]. Substantial evidence indicates that ductal cells are the primary origin of most PDAC cases [11,23]. Ductal cells are epithelial cells that comprise 10% of the exocrine pancreas and form small tubes known as ducts [23]. Their function consists of transporting digestive enzymes from the acinar cells to the duodenum [21].

**Figure 2:**
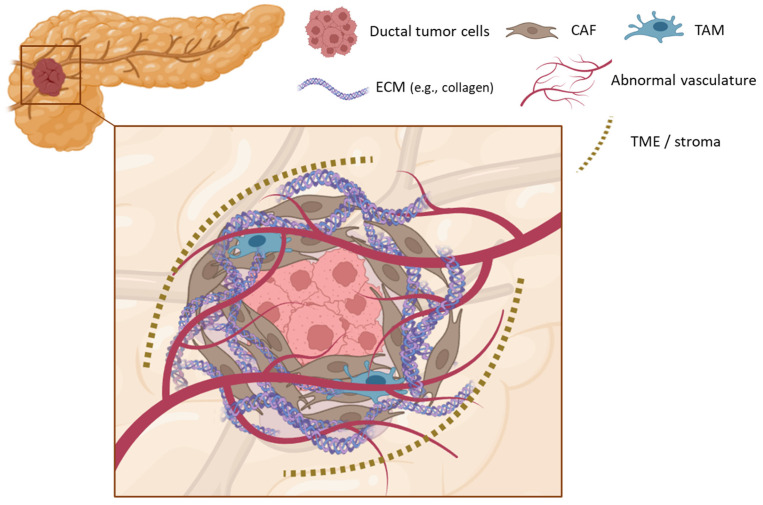
Illustration of the characteristic desmoplastic stroma of PDAC. The legend highlights ductal tumor cells, cancer-associated fibroblasts (CAFs), tumor-associated macrophages (TAMs), the extracellular matrix (ECM), abnormal vasculature, and the boundaries of the tumor microenvironment (TME). Image created using BioRender.com.

Regarding the origin of PDAC, chronic pancreatitis is a major risk factor for promoting tumorigenesis, since chronic inflammatory processes often lead to cellular and tissue changes that favor malignant transformation [24]. In this context, ductal cells eventually can undergo the EtM transition event, changing their phenotype to mesenchymal [25]. The new phenotype is characterized by higher migratory capacity, invasiveness, and high resistance to apoptosis, features which form the basis of the high metastatic potential of pancreatic cancer cells [26,27]. One hallmark of pancreatic tumors is the exuberant stroma that dominates the tumor microenvironment (TME), where stromal cells promote malignant processes such as the EtM transition by the secretion of cytokines and growth factors [24,25]. A growing body of evidence confirmed that in PDAC, the TME plays a pivotal role in tumor development, invasion, and metastasis [20,28]. The neoplastic epithelium resides within a characteristic dense stroma, which is the cellular environment in which a tumor exists, localized between the tumor and the healthy tissue [29,30]. The TME encompasses the components that are in a constant dynamic interaction with tumoral cells, involving tumor blood vessels, lymphatic vessels, the extracellular matrix (ECM), non-tumor cells such as cancer-associated fibroblasts (CAFs), and secreted signaling molecules (Figure 2) [28,30,31].

Pancreatic stellate cells (PSC) are resident cells present in the exocrine portion of the pancreas, possessing a broad range of properties, including contractility, environmental sensing through specialized cell extensions, and the elaboration of ECM components [32]. During malignant processes, these PSCs can be activated into CAF [33]. This activation has been hypothesized to be driven by cross-talk between cancer cells and PSCs, mediated by signaling molecules including the sonic hedgehog protein, the cytokines TGF-β, TNF-α, and interleukins 1, 6, and 10 [34,35]. Additionally, evidence suggests that other signaling processes can reprogram stromal cells, such as direct cell-to-cell contact, specifically methylating the DNA of specific genes [36]. Once activated, CAFs, stimulated by pancreatic cancer cells, are the main contributors of desmoplasia through their excessive proliferation and the upregulation of ECM proteins, involving fibronectin, laminin, hyaluronic acid (HA), proteoglycans, tenascin C, and, especially, collagen [24,30]. The substantial increase in the deposition of these structural components results in a dense ECM, which forms the stroma and occupies the bulk of the tumor mass [24,30,37]. Intriguingly, other cells such as macrophages have been found to play a key role in promoting fibrosis and ECM remodeling, even regulating immune suppression. These cells are called tumor-associated macrophages (TAMs) and contribute to chronic inflammation, angiogenesis, metastasis, and drug-resistance [38,39]. Furthermore, enhanced angiogenesis is present in these tumors, leading to chaotic vasculature. This tumor-associated vasculature is composed of leaky vessels with blind ends, shunts, and a tendency to collapse. These abnormalities contribute to the low and unstable oxygenation within the TME [40]. Paradoxically, this hypoxic stromal environment promotes both tumor growth and metastatic spread while also inducing vascular collapse, thereby creating a barrier to the delivery of therapeutic agents [37]. Interestingly, although this process isolates malignant cells, it was discovered that the ECM still provides them with essential amino acids required for their invasive growth [41].

## 3. Conventional Treatments in PDAC

Traditionally, cancer treatments have focused on targeting neoplastic cells, employing methods such as surgery, radiation, chemotherapy, hormone therapy, and immunotherapy to address the rapidly proliferating tumor cells [42]. The first recommended therapeutic strategy, in solid tumors, involves their surgical resection, often followed by co-adjuvant treatments such as radiotherapy or chemotherapy. Although surgery is the most effective in early stages of cancer diseases, it is not always an option, especially in advanced cancers [42,43]. Indeed, curative surgery in PDAC is still inadequate, as the recurrence rate for resected cases is approximately 87% [44]. Under these circumstances, chemotherapy remains the most widely used treatment. It is currently considered one of the most effective approaches for cancer treatment, offering a diverse range of chemotherapeutic agents with varied mechanisms of action. However, despite advances in chemotherapy, the effectiveness of treatment in PDAC remains severely limited by multidrug resistance (MDR), driven by genetic heterogeneity and a fibrotic stromal environment, underscoring the need for a deeper insight into these complexities to enhance therapeutic outcomes [9,31].

### 3.1. Limitations of Conventional Treatments

MDR represents a major obstacle to effective therapeutic approaches against cancer. In this context, MDR refers to the ability of cancer cells to tolerate anticancer agents, significantly reducing the effectiveness of therapies and contributing to treatment failure in over 90% of patients with metastatic cancer [5,27,45]. Tumors can either be intrinsically resistant to specific drugs or develop resistance during treatment [16]. It has been found that the genetic diversity in human tumors leads to the rapid emergence of drug-resistant cells in response to the pressure exerted by toxic treatments [46].

**Figure 3:**
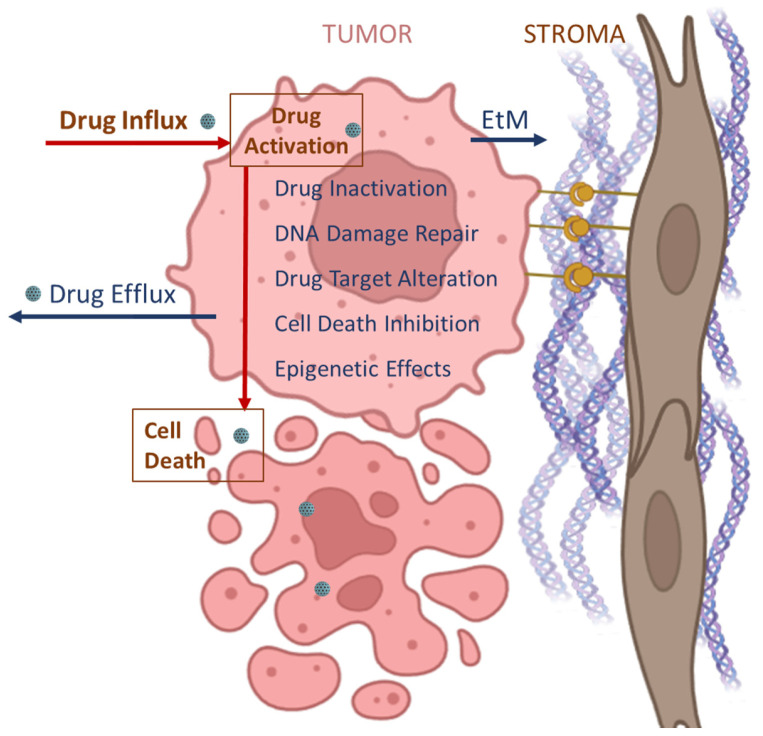
Depiction of categories that promote direct or indirect MDR in human cancer cells within a TME, highlighted in blue. The pathway leading to cancer cell death is shown in red. TME: tumor microenvironment; EtM: epithelial-to-mesenchymal transition. Adapted from the literature [27]. Image created using BioRender.com.

Drug efflux is one of the most extensively studied mechanisms of MDR, leading to the enhanced export of therapeutic agents from tumor cells (Figure 3) [27,45]. Regarding PDAC, both the EtM transition and the TME are critical factors that facilitate MDR. Although the role of the EtM transition in cancer resistance is still an emerging area of research, it may be influenced by processes involved in cell differentiation. The transition to a mesenchymal phenotype is a mechanism by which cancer cells from solid tumors become metastatic while enhancing their survival. This process is known to be regulated by both cancer and stromal cells. Moreover, the interaction of cancer cells with stromal cells and ECM elements are known to contribute the other forms of resistance, for example, by influencing epigenetic factors of cancer cells [27]. Furthermore, stromal cells not only hinder drug internalization but also secrete growth factors that stimulates cell proliferation and prevent the clearance of tumor cells [16,27,47].

### 3.2. Evolution of Chemotherapy in PDAC Treatment

**Scheme 1:**
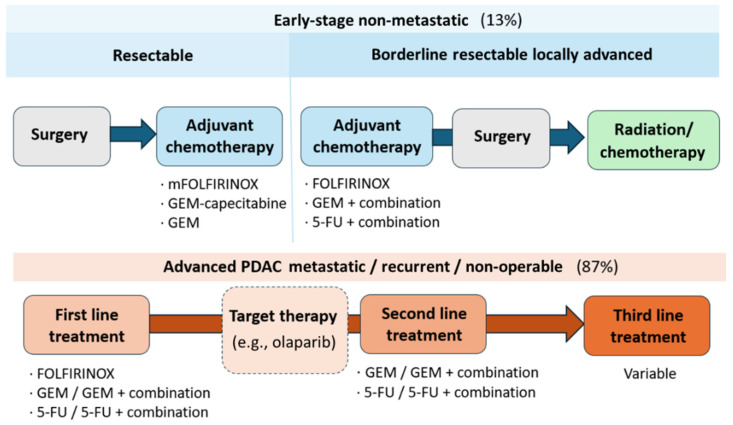
General therapeutic approaches for PDAC categorized by disease stage. Percentages indicate the proportion of patients diagnosed at each stage. Clinical approaches to advanced PDAC are discussed in more detail in the following section. Adapted from the literature [51].

In 1996, gemcitabine (GEM) monotherapy was established as the gold standard for the treatment of PDAC across all disease stages. GEM demonstrated superior efficacy and a more favorable safety profile compared to the previously used chemotherapeutic agent, 5-fluorouracil (5-FU). Despite initial clinical benefits, the development of MDR, typically within a few months of treatment, significantly limited the long-term efficacy of GEM [15,48]. To overcome the limitations imposed by MDR, combination therapy, employing multiple mechanisms of action, has been recognized as a cornerstone of cancer treatment. Thus, GEM has been used in combination with other chemotherapeutic agents, including capecitabine, paclitaxel (PTX), and oxaliplatin [9]. However, these combinations have failed to demonstrated a significant improvement in the treatment efficacy [15,49]. A major breakthrough came in 2011 with the introduction of FOLFIRINOX, a combination of folinic acid, also known as leucovorin (LV), oxaliplatin, irinotecan (IRI), and 5-FU. This combination therapy exhibited a substantial improvement in clinical outcomes, doubling the median overall survival compared to GEM monotherapy, and it has been established as the new first-line chemotherapy for PDAC (Scheme 1) [8]. However, due to the toxic character of these treatments, modifications have been made to mitigate side effects in less tolerant patients. To address these concerns, modified regimens such as mFOLFIRINOX have been developed, typically involving dose reductions in IRI. Another alternative, FOLFOX, comprising only folinic acid, oxaliplatin, and 5-FU, excludes IRI and presents a more tolerable option for patients who may not withstand the FOLFIRINOX regimen, while exhibiting significant cytotoxic effects [49,50].

Over the past decade, various therapies for PDAC have largely failed. For example, while numerous immune-based therapies have been approved for other cancers, none have been approved for pancreatic cancer due to its immunosuppressive TME, which shields tumor cells and hinders effective immunotherapy [15]. Targeted therapies, however, offer promise for specific mutations in PDAC genomes. Kinase inhibitors targeting growth factors have become prevalent due to their success in treating other cancers. In PDAC, only erlotinib has shown minimally significant improved efficacy over GEM monotherapy, earning FDA approval for metastatic, non-operable PDAC in combination with GEM [52]. Additionally, antibody-based targeted therapies, such as olaparib and pembrolizumab, have been approved for specific mutations affecting fewer than 10% of patients. Table 1 summarizes the main drugs approved for PDAC treatment in patients and their mechanisms of actions.

## 4. Nanomedicines as Emergent Treatment for Cancer

Despite advances in treating PDAC, conventional chemotherapeutics are limited by severe side effects, low accumulation in target tissues, rapid metabolism, and emerging drug resistance [42,59,60]. The alarming high mortality rate of PDAC highlights an urgent need for improved early-detection methods and more effective therapeutic strategies [49,59]. Addressing the challenging TME in PDAC would require the development of advanced, controlled drug release therapies that can precisely target the elusive pancreatic malignant cells while minimizing side effects [61].

Over the past few decades, nanomedicine has emerged as a promising discipline to address some of the limitations of conventional cancer treatments. Nanomedicine involves the application of nanoscale materials and devices to develop DDSs that enable controlled drug release. These nanoscale carriers, known as nanoparticles (NPs), possess unique properties, such as a high surface-to-mass ratio and the ability to adsorb and transport other substances [52,53,54]. Although nanomedicine is still in its early stages, nanoformulations have become a leading strategy for cancer imaging and therapy, with over 20 formulations approved for treating various cancers [31,62,63,64]. Integrating nanoscience into cancer treatments offers several advantages over conventional therapies, including controlled and sustained drug release, reduced systemic toxicity, enhanced stability, and increased drug concentration at the target site [62,65,66,67,68].

**Figure 4:**
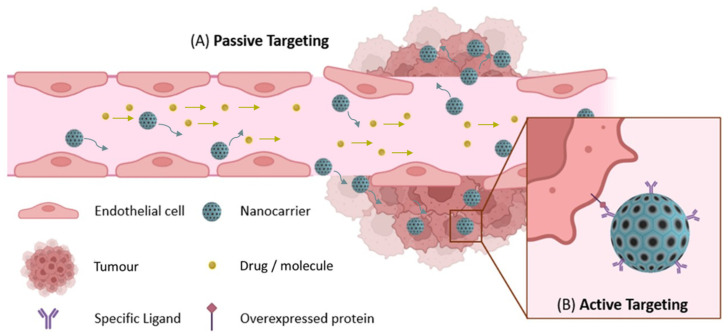
Graphic representation of an abnormal blood vessel and the transport of both small molecules and NPs through the bloodstream. (**A**) EPR effect; (**B**) selective targeting using specific ligands. Image created using Biorender.com.

The abnormal TME in cancers exhibits distinct characteristics, such as irregular vasculature (Figure 4A), low pH levels, and elevated concentrations of the reductant glutathione (GSH) in the cytoplasm of cancer cells [20]. These key features can be leveraged to enhance the accumulation of DDSs and the release of drugs from nanocarriers within the TME through the design of selective stimuli-responsive systems [11,31,69]. To optimize their accumulation, it is well established that nanocarriers for cancer therapy should have hydrodynamic sizes ranging from 60 to 200 nm to exploit the enhanced permeability and retention (EPR) effect. This phenomenon, illustrated in Figure 4A, results from the abnormal, leaky vasculature of tumors. This size range enables preferential accumulation at the target site through the EPR effect, a mechanism not effective with conventional small-molecule drug treatments [70,71,72,73,74,75,76,77].

## 5. Emerging Nanosystems in PDAC Therapy

**Figure 5:**
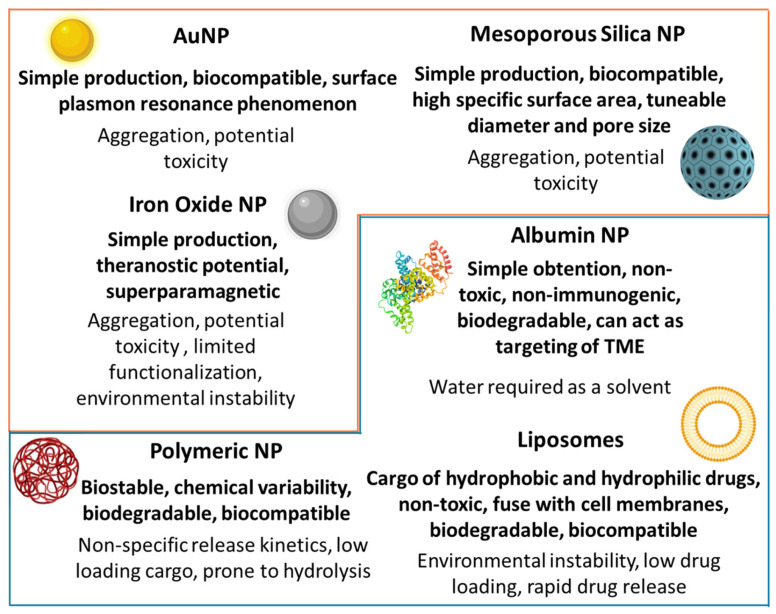
Overview of the principal nanoparticles (NPs) used in the development of DDSs for chemotherapy in PDAC. In orange: inorganic NPs; in blue: organic NPs. Advantageous properties are highlighted in bold, while negative features are presented in regular text. Image created using Biorender.com.

The highly metastatic and desmoplastic stroma of PDAC presents unique challenges that introduce additional hallmarks and treatment strategies to consider, such as the overexpression of specific proteins in either stromal or tumor cells, enabling specific active targeting strategies (Figure 4B) [69,78]. Various types of nanoparticles have been utilized to develop promising nanosystems to enhance the therapeutic efficacy of drugs for the treatment of PDAC (Figure 5). The present section provides a comprehensive overview of the nanosystems reported in the literature for PDAC. Specifically, Table 2, Table 3 and Table 4 summarize these systems and point out their main features.

### 5.1. Monotherapy Nanocarriers for PDAC Treatment

Monotherapy nanocarriers refer to nanosystems designed to deliver a single therapeutic agent with the aim of improving its pharmacological properties, bioavailability, and tumor-targeting ability of an individual anticancer agent. Table 2 compiles nanoconstructs developed for PDAC treatment that encapsulate one drug.

**Figure 6:**
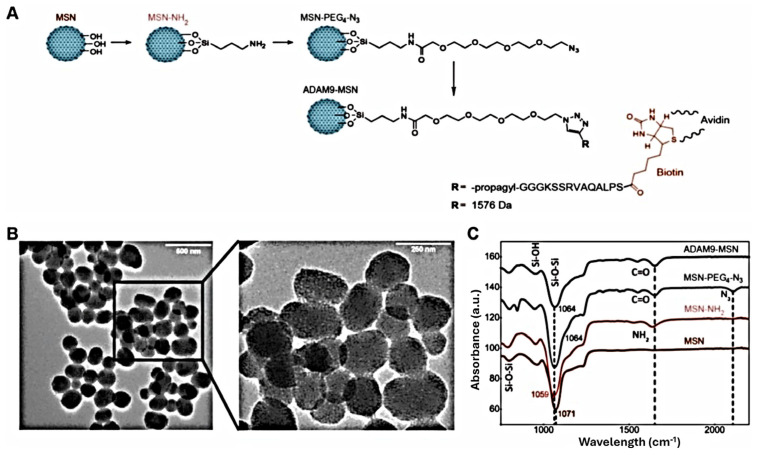
Synthesis and characterization of ADAM9-MSNs. (**A**) Schematic overview of the stepwise synthesis of peptide–biotin-coupled MSNs. (**B**) Transmission electron microscopy images of peptide–biotin-coupled MSNs. (**C**) Fourier-transform infrared spectra. Reproduced from https://doi.org/10.3390/cancers13133321. Copyright ^©^ 2021 by Arnold Spek et al. [80].

It is well-known that some chemotherapeutic agents such as paclitaxel (PTX), irinotecan (IRI) and cisplatin (CisPt) exhibit high water hydrophobicity, hindering their administration and delivery to target cells. This challenge can be exemplified by PTX, a key component of FOLFIRINOX gold standard therapy. Many authors have developed carriers to address paclitaxel hydrophobicity, involving albumin NP [11], polymeric NPs [79], and MSNs [80,81,82]. These systems have demonstrated controlled and sustained release, and some of them presented enhanced selectivity and cytotoxicity in *in vivo* assays, such as matrix metalloproteinase (MMP)-responsive systems (Figure 6) [80,81]. Particularly, the conjugation of PTX with albumin, known as nab-paclitaxel (nab-PTX, Abraxane^®^), has become the proof of concept for the development of nanomedicines for PDAC therapy [83]. This nanoformulation received FDA approval in 2013 for its use, in combination with GEM, as a frontline therapy for patients with advanced-stage metastatic PDAC [11]. Moreover, the outcomes suggested that albumin not only overcomes the poor solubility limitation of PTX, but it also acts as a targeting agent for the TME [15,78,84]. This is attributed to its affinity for the secreted protein acidic and rich in cysteine (SPARC), overexpressed by stromal fibroblast [78], and glycoprotein60 (GP60), an albumin receptor on endothelial cells that facilitates albumin transport across vascular cell layers [85].

Among the various types of nanoparticles (Figure 5), organic formulations, in particular liposomes, are the most commonly used DDSs in medicine. Liposomes are vesicles composed of phospholipid bilayers capable of encapsulating both hydrophilic and hydrophobic drugs. These types of formulations are the most FDA-approved platforms for cancer drug delivery due to their excellent biocompatibility [61,78,86].

In 2015, nanomedicine MM-398 (nal-IRI) was approved by FDA as a second-line therapy for metastatic PDAC in combination with 5-FU and LV [84]. Nal-IRI is a liposomal NP about 111 nm in diameter, encapsulating irinotecan. This formulation achieved high drug loading while optimizing retention and drug delivery in the tumor in comparison with the free drug IRI [18]. Furthermore, to enhance its therapeutic potential, this approach was improved by co-administering nal-IRI with 5-FU, leucovorin, and oxaliplatin, resulting in the formulation known as NALIRIFOX. The clinical evaluation of NALIRIFOX, using nab-PTX as the reference treatment, resulted in FDA approval in February 2024 as a new first-line treatment for advanced metastatic PDAC [12,87].

Over the past decade, research into inorganic nanoparticles for targeted PDAC nanocarriers has grown substantially. Among these, mesoporous silica nanoparticles (MSNs) have gained prominence due to their versatility and significant potential for surface functionalization [78,88]. Notably, hybrid systems, incorporating both organic and inorganic nanoparticles, have attracted significant attention due to their ability to combine the advantages of both types of materials [89,90,91,92]. Included in this group, lipid-hybrid MSNs are notable because they effectively integrate the biocompatibility of liposomes with the porosity and high loading capacity of MSNs [93,94]. For instance, Meng and colleagues demonstrated enhanced potency of IRI [93,94], oxaliplatin, and CisPt [95] by customizing lipid bilayer-modified MSNs capable of carrying high quantities of each drug while resulting in minimal leakage. Their DDSs demonstrated equivalent or superior biocompatibility and therapeutic efficacy compared to liposomal formulations and free drug assays in preclinical studies [93,94,95]. Moreover, the same authors used the lipid-film-coated procedure to rapidly seal GEM in a hybrid lipid MSN [96], improving the outcomes of their previous systems [97].

It should be noted that GEM is still a crucial drug in most available treatments for patients, either in combination or as a monotherapy (Scheme 1). Since GEM is highly water-soluble, its loading into certain carriers [89,98,99] and diffusion into cells are compromised [84]. However, this drawback has been addressed by other researchers through the attachment of GEM onto functionalized MSNs [100] or by utilizing more compatible carriers, such as albumin [101], liposomes [87,102,103], and polymeric NPs [72,73,104,105]. These strategies enhanced cellular internalization, improving the therapeutic efficacy of free GEM treatments.

In addition to the chemotherapeutic agents commonly used in PDAC treatment, other drugs with limited clinical application due to their hydrophobicity and poor biodistribution could become effective strategies when encapsulated in NPs. For instance, curcumin, loaded into liposomes [106], polymeric NPs [107], and MSNs [108], has been shown to inhibit tumor growth and reduce metastasis in PDAC xenograft models [78,106,108]. Similarly, the use of camptothecin (CPT) in clinics, the natural precursor of IRI, is also limited by its hydrophobicity and the significant side effects it causes. However, when encapsulated in nanocarriers, CPT has demonstrated substantial tumor regression in PDAC models using MSNs [109,110] and polymeric NPs [111], in comparison to the standard first-line drug GEM, for advanced pancreatic cancer [111].

**Table 2:** Summary of the main DDSs used for PDAC monotherapy. Glutathione (GSH); hydrodynamic size (HS); mesoporous silica NP (MSN); nanoparticle (NP); photodynamic therapy (PDT); polyethylene glycol (PEG); arginylglycylaspartic acid (RGD); reactive oxygen species (ROS); real size (RS); sonodynamic therapy (SDT). Targeting molecules are underscored for clarity.

Drug	Formulation	Nanocarrier	Size (nm)	Characteristics	Ref.
PTX paclitaxel	nab-PTX	Albumin NP	130 RS	TME Targeting	[83]
	PTX@MSN-responsive-ADAM9–biotyn–avidin	MSN	189 HS	Avidin-capping Protease-responsive linker	[80]
	PTX@MSN-responsive-CAPN2–biotyn–avidin	MSN	235 HS	Avidin-capping Protease-responsive linker	[81]
	PLGA-PTX	Polymeric NP	160 HS	pH-sensitive polymeric coating	[79]
	PTX@MSN	MSN	100 RS	-	[82]
IRI irinotecan	nal-IRI/MM-398	Liposomal NP	111 RS	-	[84]
	Liposome conjugated to MSN	Hybrid lipid MSN	80 RS 100–150 HS	Lipid bilayer	[93]
	Liposome conjugated to MSN with Au core	Hybrid lipid MSN	130 HS	Lipid bilayer	[94]
GEM gemcitabine	RGD peptide-conjugated magnetic MSN	Magnetic modified MSN	50 RS	Tumor-targeting	[89]
	Liposome–exosome fusion	Liposome	<200 HS	Tumor-targeting Increased uptake	[87]
	Liposome PEGylation-ligand	Liposome	<100 RS	Tumor-targeting	[102]
	Flow Focusing^®^	Polymeric NP	655 HS	High payload Narrow size distribution	[72]
	MSN	MSN	42–64 RS	Pore-expanded	[99]
	HSA-GEM/IR780	Albumin	<10 HS	Cleavable peptide cathepsin B Imaging with IR780	[101]
Curcumin	Curcumin@PEGylated MSN-Transferrin	MSN	120 RS/167 HS	PEGylation Tumor-targeting	[108]
	Liposomal curcumin	Liposome	-	pH-responsive	[106]
	NanoCurc™	Polymeric NP	50 RS	pH-responsive	[107]
Methylene Blue	Gold-NP attached organically MSN	Au-MSN hybrid NP	30/55/80 RS	PDT	[90]
CisPt cisplatin	Iron oxide NP covered silica shell -CisPt	Magnetic- MSN	54 RS	pH-responsive	[91]
	Liposome conjugated to MSN	Hybrid lipid MSN	82 RS 137 HS	pH-responsive	[95]
FdUMP 5-FU metabolite	Aptamer (CCK-B)-PEG-FdUMP-CPNs	Calcium phosphosilicate NPs (CPN)	<100 RS	Tumor-targeting pH-sensitive	[112]
CPT camptothecin	CPT@MSN–Folate modification	MSN	100–150 RS	Tumor-targeting	[109]
	Dendrimers–CPT Charge switchable	Polymeric NP	26 RS	ROS-responsive linker Increased uptake	[111]
	aptamer/cell-penetrating peptide–camptothecin prodrug NPs	camptothecin prodrug NPs	131 HS	Tumor-targeting	[113]
DOX doxorubicin	DOX liposome Charge switchable	Liposome	65 HS	GSH-responsive Increased uptake	[92]
	Fluorinated amphiphilic dendrimer	Micelle	10 RS	Self-assembly micelles	[114]
Ce6 chlorin e6	Polyphosphoester-based nanocarrier	Polymeric NP	40 RS	pH-responsive evasion immune clearance	[115]
Benzoporphyrin derivative	Eutectic gallium–indium NPs, gallium oxide shell Conjugated to hyaluronic acid	Gallium-indium NPs	25–65 RS	PDT	[116]
IRT80	Encapsulated fluorocarbon chains and IRT80	Hollow MSN	<200 RS	Oxygen delivery, SDT	[117]
Rose bengal	Sulfur hexafluoride PEG–biotin–avidin	Lipid microbubbles	1–2 μm	Oxygen delivery, SDT	[118]

Remarkably, photodynamic therapy (PDT) has emerged as a promising two-stage treatment based on the selective damage of tumoral cells, microorganisms, and blood vessels. This is achieved through the administration of a photosensitizer (PS), a non-toxic drug able to be selective while activated by light generating reactive oxygen species (ROS) [119]. Nanocarriers based on PDT have been developed for pancreatic cancer due to its high cytotoxic potential, achieving cell viabilities as low as 5%, thereby outperforming other therapies in targeting tumor cells [90,115,116,117,120,121]. Unfortunately, the fibrotic stroma and abnormal vasculature of PDAC contribute to severe hypoxia. Oxygen levels in PDAC tissue are estimated to be roughly 0.3%, compared to approximately 7.5% in healthy pancreatic tissue, constraining the PDT effectivity in pancreatic tumor cells [122]. In hypoxic environments the low oxygen levels hinders the formation of ROS. Nevertheless, the high cytotoxicity demonstrated by PDT in cancer cells has motivated researchers to design oxygen delivery strategies to overcome this challenge. To mitigate the hypoxia of PDAC tumors, some authors used AuNPs to increase the cytotoxic potential of ^1^O_2_. This effect is achieved by leveraging the localized surface plasmon resonance phenomenon of AuNPs, which facilitated surface energy transfer between the PS molecules and NPs. Such carriers are known to extend the lifetime of ^1^O_2_ through this mechanism, thereby improving the efficacy of PDT even under hypoxic conditions [90].

Other authors developed systems able to deliver oxygen to the target site by modification of carriers with fluorocarbon chains, increasing the efficacy of sonodynamic therapy (SDT), a variant of PDT, since it was demonstrated that several PS can also be activated by ultrasound irradiation. It has been demonstrated that several PSs can also be activated by ultrasound, a strategy that offers significant advantages over PDT, including cost-effectiveness, safety as a clinical imaging modality, and tunable penetration depending on the frequency used [117,118].

### 5.2. Combination Therapy Nanocarriers for PDAC Treatment

**Scheme 2:**
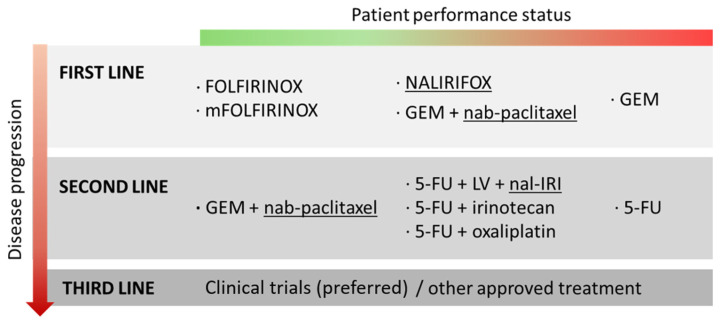
Overview of current approved treatments for metastatic PDAC patient therapy [12,51]. Treatments involving NPs are underlined. GEM: gemcitabine; 5-FU: 5-fluorouracil; LV: leucovorin, NALIRIFOX: nal-IRI + 5-FU + LV+ oxaliplatin; FOLFIRINOX: 5-FU + LV + irinotecan + paclitaxel; mFOLFIRINOX: modified-FOLFIRINOX.

As depicted in Scheme 2, breakthroughs in nanotechnology are significantly redefining the therapeutic landscape for metastatic PDAC. Independent preclinical and clinical studies of nab-PTX and nal-IRI have proved that these nanoformulations offer superior pharmacokinetic profiles compared to their free drug counterparts. However, these formulations are not effective as standalone therapies; their combination with established antineoplastic agents is critical for achieving significant therapeutic efficacy and presenting notable improvements over conventional treatment strategies for PDAC [18,84,123]. These findings underscore the importance of combination therapy in the development of effective PDAC treatments; thereby, the co-delivery of multiple drugs in a single carrier emerges as a promising framework against this challenging disease.

Nanocarriers for combination therapies are engineered to co-deliver two or more therapeutic agents within a single nanosystem, aiming to enhance treatment efficacy through combining mechanisms of action. This section explores the design, advantages, and therapeutic outcomes of combination nanocarrier systems developed for PDAC therapy (Table 3).

**Table 3:** Primary DDSs for the co-delivery of drugs for PDAC treatment. Chemo refers to chemotherapy. Cisplatin (CisPt); camptothecin (CPT); doxorubicin (DOX); gemcitabine (GEM); glypican 1 (GPC1); glutathione (GSH); hydrodynamic size (HS); mesoporous silica NP (MSN); glycoprotein mucin 1 (MUC1); nanoparticle (NP); photodynamic therapy (PDT); polyethylene glycol (PEG); polyethylenimine (PEI); poly(lactide-co-glycolide) (PGLA); paclitaxel (PTX); real size (RS); sonodynamic therapy (SDT) zinc-phthalocyanine (Zn-Pc).

Drugs	Therapy	Nanocarrier	Composition	Size (nm)	Features	Ref.
GEM/PTX	Chemo	Polymeric NP	Tri-block co-polymer tumor-targeted peptide	159 HS	pH-responsive tumor-targeted Inhibition GEM deactivation	[104]
	Chemo	Hybrid lipid polymeric NPs	Lipid-bilayer PGLA	70 HS	pH-sensitive Drug conjugate Inhibition GEM deactivation	[124]
	Chemo	Hybrid lipid MSNs	Lipid bilayer	65 RS	pH-responsive	[96]
GEM/CisPt	Chemo	Antibody (TAB004)-GEM-CisPt-MSN	PEI/PEG	150–200 HS	Redox-responsive Tumor-targeting	[100]
GEM/iron oxide NPs	Chemo, Thermal	Polymeric NPs	PLGA HER-2 Antibody	534 HS	Tumor-targeting	[73]
GEM/Iron oxide NPs/Cetuximab	Thermal, Chemo, Targeted	Magnetic Albumin NP	Iron oxide NPs Cetuximab	200 HS	Tumor-targeting Imaging	[125]
GEM/Au-NP	Chemo, PDT, Thermal	Gold-nanoshell-coated MSN	Transferrin Gold-nanoshell	100–150 RS	Tumor-targeting	[120]
GEM/Ferulic acid	Chemo, Antioxidant	MSN	anti-GPC1 antibodies	206 HS	Tumor-targeting Low cargo	[98]
GEM/ONC201	Chemo, Targeted	Liposomes	ONC201 (MUC1 peptide)	113 HS	Tumor-targeting Apoptosis upregulation	[103]
Oxaliplatin/Indoximod	Chemo, Immune	Hybrid lipid MSN	Lipid bilayer	83 RS	Immunoactivator	[126]
Bortezomib/IR-820	Chemo, PDT, thermal	Hybrid lipid MSN	Ciclosporin A	160	Increased uptake	[121]
Zn-Pc/Cetuximab	PDT, Targeted	MSN	PEGylated Cetuximab	303 HS 79 RS	Tumor-targeting	[74] [75]
CPT/DOX	Chemo	MSN	Quantum dot	150–200 RS	pH-responsive	[110]
DOX/hydroxychloroquine	Chemo	Mesoporous silica nanorods	-	180 × 60 RS	Autophagy inhibition Macropinocytosis selectivity	[127]
DOX/iron oxide NP	Chemo, Thermal	Magnetic-MSN	Shell of MSN	55 RS 106 HS	Thermal-sensitive caps	[128]

Numerous nanocarriers developed for PDAC have been based on combining chemotherapeutic agents, aiming to mitigate the common MDR associated with chemotherapeutic agents like GEM. For instance, PTX has been reported to function as an inhibitor of cytidine deaminase, the enzyme responsible for deactivating GEM, highlighting the significant therapeutic potential of combining PTX and GEM. Specifically, polymeric [104], hybrid lipid polymeric [124], and hybrid lipid MSN [96] formulations exhibited high stability and synergistic cytotoxic effect in mouse xenograft models of PDAC. As mentioned, Meng and colleagues developed a lipid film-coated method to rapidly encapsulate GEM in a hybrid lipid–MSN system. This lipid entrapment not only significantly increased GEM’s encapsulation efficiency but also enabled the co-loading of paclitaxel. As a result, the system showed enhanced efficacy compared to nab-paclitaxel combined with free GEM in preclinical studies [96]. Furthermore, Zhang and coworkers synthesized a GEM-PTX conjugate (1:1) via hydrolysable linker to encapsulate it in hybrid lipid polymeric NPs [124]. The conjugation through a sensitive linker endows the system with even more selectivity, minimizing size effects. The cytotoxicity of the resulting GEM-PTX against pancreatic cancer cells not only was comparable to that of the corresponding free drug mixtures, but also was significantly improved after its encapsulation into hybrid lipid polymeric NPs [124]. Interestingly, although drug conjugation offers superior control over drug stoichiometry and release kinetics, this strategy remains mainly underexplored in drug delivery approaches for PDAC, as observed in Table 3.

Other authors have taken advantage of the aforementioned high specific surface area of MSNs to design advanced drug delivery systems capable of co-loading multiple therapeutic agents within a single carrier [68,88,129]. Escoto and colleagues attached GEM and CisPt onto MSN surfaces thorugh a redox-sensitive linker and found cytotoxic synergistic effect with their system [100]. Moreover, the presence of polyethyleneimine (PEI) and polyethylene glycol (PEG) polymers on the NP surfaces increase its uptake and lifetime through the bloodstream, respectively. In addition, the conjugation of a specific antibody to the PEG chains endows the system with active targeting, further enhancing the internalization of the system to pancreatic tumoral cells [100].

To further improve the efficacy of anticancer drugs, chemotherapeutic agents have also been combined with other active molecules and therapies to explore other ways to tackle PDAC, including antioxidants [98], targeted therapy [103], thermal therapy [73], immunomodulation [126], autophagy inhibition approach [127], and photodynamic therapy [121].

In 2017, Meng and colleagues were the first to demonstrate an immune-based approach for drug delivery in PDAC. Indoximod was selected to activate the immune system by the suppression of an immunosuppressive pathway. Moreover, the co-delivery of this immune modulator with oxaliplatin resulted in a synergistic immune response in preclinical models [126].

**Figure 7:**
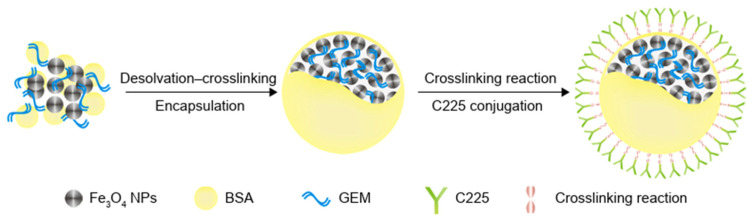
Schematic procedure for synthesis of C225-GEM/MANs. BSA: bovine serum albumin; C225: cetuximab; GEM: gemcitabine; MANs: magnetic albumin nanospheres; NPs: nanoparticles. Reproduced from reference https://doi.org/10.2147/IJN.S77642. Copyright ^©^ 2015 Taylor & Francis Group [125].

Regarding targeted therapy, the co-delivery of GEM and cetuximab via magnetic-albumin NPs permits not only the triple therapy by magnetic-targeted thermo-chemotherapy but also the targeting of both stromal and tumor cells. While albumin facilitates the retention of the system at the TME, cetuximab targets the cancer cells, further enhancing the system’s uptake by PDAC cells (Figure 7) [125]. Another example is the combination of GEM with the novel targeted molecule ONC201. This combination therapy was encapsulated in a liposomal formulation, resulting in the upregulation of apoptosis and enhanced accumulation of the carrier within the TME. However, preclinical studies demonstrated no significant advantages beyond the inhibition of tumor progression [103].

PDT was also exploited in combination with chemotherapeutic agents. For instance, Nie and coworkers introduced a hybrid lipid MSN system for triple therapy, combining chemotherapy with bortezomib, photothermal therapy using IR-820, and enhanced cell penetration via cyclosporine A [120]. Another example is given by Yurt and colleagues. The authors prepared PEGylated MSNs loaded with zinc phthalocyanine and conjugated with cetuximab for combination therapy of PDT and targeted therapy against a growth factor receptor demonstrated limited effectiveness in preclinical experiments [75].

It should be noted that in recent examples, active targeting has played a major role in enhancing the uptake of nanocarriers by pancreatic cancer cells [103,125]. Many of the systems summarized in Table 2, Table 3 and Table 4 are functionalized with various targeting molecules, including liposomes [103], hybrid-NPs [89,120,124,125], polymeric NP [73], iron oxide NP [130], and MSN [74,98,108], which demonstrate improved selective internalization. It is also worth noting that alternative strategies, such as surface decoration with cationic or hydrophobic molecules, have been employed to increase cellular internalization. For instance, surface modification of MSNs with the cationic polymer PEI [100] and the use of charge-switchable approach in liposomes [92] and polymeric NPs [111] has proven effective in enhancing uptake in these systems [92,111]. Another example is provided by Kim and colleagues, who utilized cyclosporine A in a hybrid lipid-MSN system to improve cellular uptake in PDAC cells. This enhancement was achieved through the interaction of a hydrophobic peptide with cell membranes, promoting a more efficient internalization [121].

### 5.3. Nanocarriers for Combined Anticancer and Stromal Therapies in PDAC Treatment

The fibrotic stroma is rich in biopolymers such as hyaluronic acid and collagen that contribute to elevate the interstitial fluid pressure, which, in turn, hampers diffusion through the extracellular matrix, especially for larger-size nanocarriers [131]. Despite the advantages of combination therapies and targeting strategies to increase the intracellular internalization of nanoplatforms, this formidable barrier significantly hampers the selectivity of nano-formulations to be accumulated in the TME, contributing to the poor prognosis in PDAC treatment [37,132].

In light of this impediment, stroma modulation strategies have been emerging as attractive strategies (Table 4) [15,133]. These innovations aim to alleviate the stromal barrier, thereby enhancing drug penetration and improving the therapeutic efficacy of loaded chemotherapeutics in the treatment of PDAC [15,134].

**Table 4:** DDS for enhanced stromal penetration approaches against PDAC. Dendrigraft poly-l-lysine (DGL); docetaxel (DTX); extracellular matrix (ECM), hydrodynamic size (HS); gemcitabine (GEM); glutathione (GSH); doxorubicin (DOX); mesoporous silica NP (MSN); nanoparticle (NP); polyethylene glycol (PEG); polyethylenimine (PEI); poly(lactide-co-glycolide) (PGLA); poly(ethylene glycol)-poly(caprolactone) (PP); plectin-1 targeted peptide (PTP); real size (RS); transforming grow factor (TGF).

Nanocarrier	Modifications	Tumor Therapy	Stromal Approach	Size (nm)	Features	Ref.
Hybrid liposome-MSN	Co-polymer coating of MSN: PEG/PEI	GEM	TGF-β inhibitor	143→50 RS	pH-responsive Low loading	[97]
MSN	DOX@MSN-*S*-nitrosothiol/PEG	DOX	*S*-nitrosothiol	107 HS	Collagen depletion	[134]
Mesoporous polydopamine	CAF membrane, PTP	Iron carbonyl	Losartan	126 nm	ECM degradation	[135]
Lipid NPs	aPD-L1 (antibody), perfluoropentane	DTX	Pulsed ultrasound stimulation	95 nm	Antifibrotic, recover hypoxia	[136]
HMON	Hollow organically MSN	GEM	pirfenidone	115→33 RS	pH/GSH-responsive	[137]
Liposome-modified Polymeric NP	Small NP: PEG-PGLA	PTX	TGF-β inhibitor Shrinkable	155→40 RS	pH-responsive	[138]
Polymeric NP	Chloroquine phosphate	GEM	Shrinkable	125→30 HS	pH-responsive Inhibition autophagy	[105]
	Dendrigraft to PP micelle (DGL/DOX@PP)	DOX	Shrinkable	100→30 RS	MMP-responsive	[139]
	dendrigraft poly-l-lysine to PP micelle	GEM	18β-glycyrrhetinic acid Shrinkable	151→30 RS	MMP-responsive	[28]
	PLGA polymer	GEM	Simvastatin	258 RS	Mitigation of stroma pH-sensitive	[77]
Iron oxide NP	pH low insertion peptides: pHLIP	GEM	Metformin	23 HS 10 RS	Stromal depletion pH-responsive	[130]

**Figure 8:**
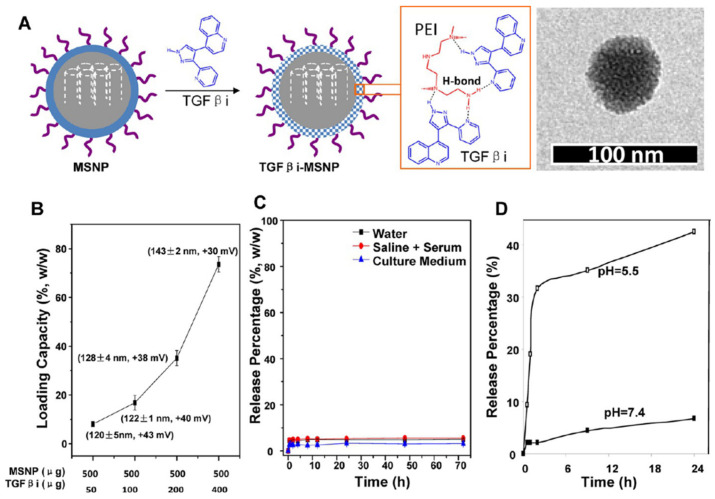
Development of an efficient TGF-β inhibitor (TGF-βi) carrier, using molecular complexation to attach TGF-βi to PEI-PEG-coated MSNP. (**A**) Graphical presentation of the particle showing H-bonding of TGF-βi to the PEI polymer. (**B**) Assessment of the maximum loading for TGF-βi to the particle. (**C**) Stability of TGF-βi attachment in different solutions. (**D**) TGF-βi release was studied in pH 5.5 aqueous solution for 24 h and compared with the release profile in PBS (pH 7.4). Reproduced from reference https://doi.org/10.1021/nn404083m. Copyright ^©^ 2013 American Chemical Society [97].

A growing number of stromal modulators have demonstrated significant potential for stromal depletion, including TGF-β inhibitors [97,138], pirfenidone [137], 18β-glycyrrhetinic acid [28], simvastatin [77], and metformin [130]. For instance, PEGylated MSNs have been employed to deliver *S*-nitrosothiol, a nitric oxide donor, aiming to activate specific proteases present in the TME that degrade collagen-composing ECM. This strategy facilitates deeper penetration of the carrier, allowing doxorubicin DOX to effectively reach the hindered pancreatic cancer cells [134]. It is worth highlighting that the two-wave nanocarrier strategy significantly enhanced tumor drug delivery (Figure 8). The first wave employed TGFβi-loaded MSNs to disrupt pericyte coverage and increase vascular permeability, priming the tumor for the second wave of PEGylated, drug-loaded liposomes. This sequential delivery enabled deeper TME penetration and superior therapeutic efficacy. By combining GEM loading with targeted TGF-β pathway inhibition, this approach achieved sustained tumor suppression beyond 25 days, outperforming both free-drug and conventional standalone liposome treatments [97].

**Figure 9:**
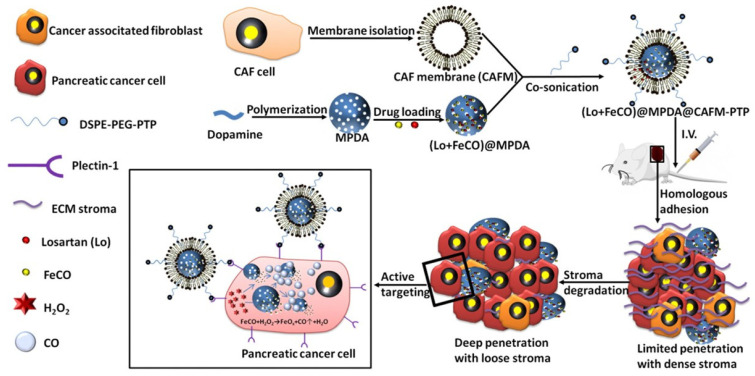
Schematic illustration of the (Lo + FeCO)@MPDA@CAFM-PTP biomimetic dual-targeting nanomedicine preparation and its therapeutic effects on pancreatic cancer through targeting CAF and pancreatic cancer cells, ECM degradation, and CO gas therapy. Losartan (Lo), mesoporous polydopamine (MPDA), cancer-associated fibroblast cell membrane (CAFM), plectin-1 targeted peptide (PTP), extracellular matrix (ECM). Reproduced from reference https://doi.org/10.1039/D4TB02206H. Copyright ^©^ 2025 Royal Society of Chemistry [135].

Recently, Cheng and colleagues engineered a nanocarrier for the dual targeting of pancreatic tumor. These researchers prepared mesoporous polydopamine nanoparticles loaded with an iron carbonyl and losartan, enabling gas therapy with CO against tumor cells and degradation of the ECM, respectively. The NPs were then coated with CAF cell membranes to facilitate the targeting of CAFs and the delivery of losartan. Additionally, functionalization of the CAF membranes with a plectin-1-targeting peptide (PTP) endowed the system with specific targeting of pancreatic cancer cells (Figure 9) [135].

The vasculature within the TME consists of tortuous, leaky, and non-uniform vessels [24]. The size of the pores or fenestrations in the tumor vascular walls range from 10 nm to 2 μm [63,131]. It is well-known that the transport of nanocarriers across tumor vessel walls and cell membranes is largely influenced by NP properties including, shape, charge, and size. For cancer targeting, NPs are typically recommended to be 20–100 nm in size [70,140]. Consequently, smaller NPs (<60 nm) are associated with greater infiltration efficiency. Paradoxically, NPs with larger hydrodynamic sizes (60–200 nm) exhibit longer blood circulation half-lives and an enhanced extravasation and retention (EPR) effect but reduced tumor permeability [70,71]. These conflicting size requirements have driven the development of size-shrinkable nanocarriers, which have emerged to balance NP accumulation and penetration in tumors simultaneously [138].

**Figure 10:**
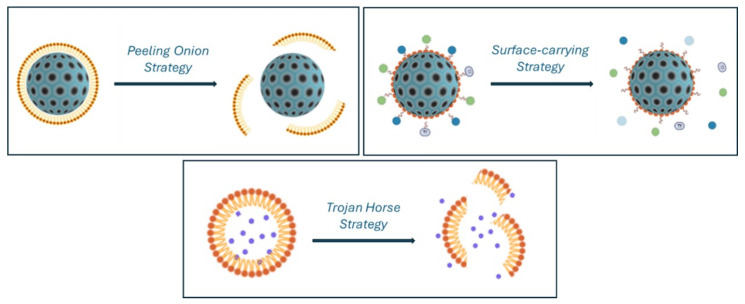
Illustration of the size-shrinkable strategies. Peeling onion strategy, surface-carrying strategy, and Trojan horse strategy. Adapted from the literature [71]. Image created using Biorender.com.

Shrinkable systems are engineered to reduce their size in response to specific stimuli, facilitating selective penetration through the stroma to target cancer cells (Figure 10). These systems can be summarized into the models according to their structural characteristics: a peeling onions strategy, a surface-carrying strategy, and a Trojan horse strategy [71]. In this context, polymeric NPs have predominantly been used to design size-switchable nanocarriers, largely due to the frequent presence of hydrolysable ester bonds [77,105]. Similarly, the incorporation of responsive linkers is a strategy commonly reported for other nanocarriers [96,113]. Furthermore, some authors have incorporated a stromal modulator into size-switchable NPs. The resulting systems exhibited enhanced drug accumulation in the TME, reduced stroma fibrosis, and deeper NP penetration, offering a promising strategy for future PDAC treatments [19,28,138].

The peeling onion strategy involves the construction of nanosystems with multilayered architecture. Each layer is designed to respond to a specific stimulus, allowing for a stepwise reduction in the overall size of the nanocarrier [71]. By that means the nanocarrier can shrink progressively through biological environments, enhancing its ability to penetrate deeper into the fibrotic stroma and target cancer cells. In this regard, NPs with versatile tunable surface and size, such as MSNs, are particularly well-suited for this strategy [88,141]. Nel and colleagues provided a notable example by developing a shrinkable co-polymer-modified MSNs, composed of PEI and PEG polymers. Once accumulated in the TME, the pH-sensitive polymer degradation reduced the size of the system from 143 nm to 50 nm while releasing LY364947, a TGF-β inhibitor [97].

**Figure 11:**
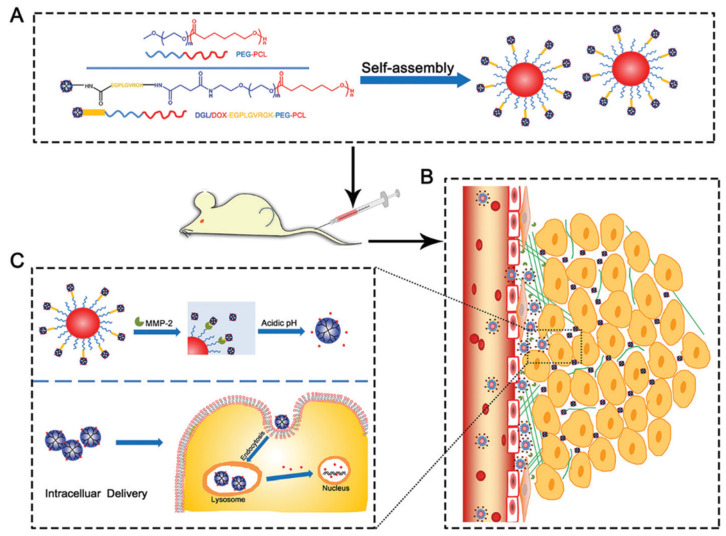
(**A**) Illustration of the preparation of MMP-2-sensitive, multistage NPs (DGL/DOX@PP). (**B**) Schematic illustration of DGL/DOX@PP accumulation at the tumor site and shrinkage to overcome the biological barriers to reach deep within the tumor. (**C**) Size reduction in response to MMP-2 in the TME and drug release in response to intracellular acidic pH. Metalloprotease (MMP), dendrigraft poly-*l*-lysine (DGL), doxorubicin (DOX), poly(ethylene glycol)-poly(caprolactone) micelles (PP), polyethylene glycol (PEG), poly(caprolactone) (PCL). Reproduced from reference https://doi.org/10.1039/C8NR00640G. Copyright ^©^ 2018 The Royal Society of Chemistry [139].

In 2018, He and colleagues developed an innovative surface-carrying strategy. This approach relies on the construction of nanocomplexes formed by small NPs tethered to the surface of a larger NP, allowing the small NPs to be released from deeper stromal penetration (Figure 10) [71]. Firstly, they constructed a system based on dendrigraft poly-*l*-lysine (DGL) conjugated to poly(ethylene glycol)-poly(caprolactone) (PP) micelles, linked via an MMP-responsive peptide. This MMP-responsive system (100 nm) was designed to release DOX conjugated to DGL by action of MMP proteins in TME. As a consequence, DGL nanocarriers of 30 nm were able to deep penetration into the stroma (Figure 11) [139]. Building on this approach, the incorporation of a stroma modulator, 18β-glycyrrhetinic acid, and autophagy inhibitor, chloroquine, have been studied in other works to further enhance therapeutic efficacy [28,105].

Another strategy, called the Trojan horse, is based on encapsulating small NPs inside large NPs, thereby enabling the release of the small NPs under certain conditions (Figure 10) [71]. Recently, Nie and colleagues pioneered a novel approach based on the Trojan horse strategy for combination of a stromal modulation strategy with a size-switchable nanosystem. PEG−PLGA nanospheres of 40 nm were carried in liposomes of 155 nm. An antifibrotic agent, a TGF-β inhibitor, was attached onto liposomal surface, while containing smaller polymeric NPs carrying PTX. Upon liposome degradation, the system delivered the stromal-modulating therapy targeting CAFs, while facilitating the deeper penetration of the smaller polymeric NPs and enables the precise delivery of PTX to tumor cells [138].

## 6. Conclusions

This review highlights the significant advantages of using nanocarriers for combination therapy in PDAC. Specifically, DDSs for multi-drug delivery can substantially reduce the systemic toxicity of chemotherapeutics, simplify treatment regimens, and enhance efficacy through optimized drug combinations. By encapsulating drugs with distinct therapeutic mechanisms into delivery systems at optimal ratios, nanocarriers enable sequential delivery tailored to clinical needs.

While the EPR effect has been a cornerstone of nanomedicine, its limitations in achieving precise targeting are increasingly evident. Nevertheless, NPs are ideal platforms for developing advanced targeting strategies for PDAC, such as incorporating ligands like peptides, aptamers, or antibodies to improve treatment selectivity. Of particular significance in the context of PDAC is the development of size-switchable nanoparticles capable of penetrating the dense extracellular matrix surrounding the tumor.

Despite the immense promise of nanocarriers, challenges such as large-scale reproducibility and long-term safety must be addressed for widespread clinical adoption. Encouragingly, several nanocarrier-based formulations have already received FDA approval, marking significant progress toward clinical translation. However, challenges persist, as FDA-approved nanocarriers for PDAC often require co-administration with conventional chemotherapeutics to achieve therapeutic efficacy comparable to standard treatments. With continued innovation, nanomedicine is poised to revolutionize pancreatic cancer therapy, offering hope for more effective and targeted treatments in the near future.

## Figures and Tables

**Table 1:** Summary of the main drugs approved for the treatment of PDAC in patients, along with their primary modes of action [15], their use (as monotherapy or combination therapy), and therapy type. Gemcitabine (GEM) [48], oxaliplatin [53], irinotecan (IRI) [54], capecitabine [55], erlotinib [52], paclitaxel (PTX) [56], 5-fluorouracil (5-FU) [57] are among the commonly used agents. Epidermal growth factor receptor (EGFR) is overexpressed in up to 89% of patients and its expression is correlated with worse outcomes [58].

Drug/Treatment	Mechanism of Action	Use	Therapy
Capecitabine	Oral prodrug of 5-FU; inhibits DNA synthesis by blocking thymidylate synthase.	Both	Chemotherapy
Erlotinib	EGFR tyrosine kinase inhibitor; blocks signaling pathways involved in cell proliferation.	Combination	Targeted therapy
Gemcitabine	Antimetabolite; inhibits DNA synthesis by incorporation into DNA and inhibition of ribonucleotide reductase.	Both	Chemotherapy
Irinotecan	Topoisomerase I inhibitor; prevents DNA unwinding, leading to DNA damage.	Combination	Chemotherapy
Oxaliplatin	Platinum-based drug; causes DNA crosslinking and subsequent apoptosis.	Combination	Chemotherapy
Paclitaxel	Binds and stabilizes microtubules, preventing cell division.	Combination	Chemotherapy
5-FU	Pyrimidine analog; inhibits thymidylate synthase, blocking DNA synthesis.	Both	Chemotherapy
FOLFOX	Combination of 5-FU (DNA synthesis inhibition), leucovorin (enhances 5-FU effect), and oxaliplatin (DNA crosslinking).	Combination	Chemotherapy
FOLFIRINOX	Combination of 5-FU (DNA synthesis inhibition), leucovorin (enhances 5-FU effect), irinotecan (topoisomerase I inhibition), and oxaliplatin (DNA crosslinking).	Combination	Chemotherapy
mFOLFIRINOX	Modified doses of FOLFIRINOX with reduced irinotecan to reduce toxicity; same mechanisms as FOLFIRINOX.	Combination	Chemotherapy
